# Growth, pectoralis muscle performance, and testis of pelung cockerels (*Gallus gallus gallus* [Linnaeus, 1758]) supplemented with blood clam shell powder (*Anadara granosa* [Linnaeus, 1758])

**DOI:** 10.14202/vetworld.2023.474-482

**Published:** 2023-03-17

**Authors:** Rizki Fitrawan Yuneldi, Claude Mona Airin, Hendry T. S. Saragih, Sarmin Sarmin, Pudji Astuti, Abdul Razak Alimon

**Affiliations:** 1 Post-Doctoral Program, Faculty of Veterinary Medicine, Universitas Gadjah Mada, Yogyakarta, Indonesia; 2 Department of Physiology, Faculty of Veterinary Medicine, Universitas Gadjah Mada, Yogyakarta, Indonesia; 3 Laboratory of Animal Development Structure, Faculty of Biology, Universitas Gadjah Mada, Yogyakarta, Indonesia; 4 Department of Animal Science, Faculty of Agriculture, Universiti Putra Malaysia, Serdang, Selangor, Malaysia

**Keywords:** growth performance, muscle, natural aromatase blocker, pelung, testis

## Abstract

**Background and Aim:**

Pelung cockerels (*Gallus gallus gallus* [Linnaeus, 1758]) are different from other native cockerels in that they have a long and unique voice, in addition to their tall, large, and sturdy body with a relatively heavy body weight (BW). The sound quality of pelung cockerels is affected by the structure of the syrinx and their large and strong chest muscles. The performance of the chest muscles, and subsequently its voice, is influenced by the hormone testosterone. The shell of blood clams (*Anadara granosa* Linnaeus, 1758), a saltwater bivalve is known to contain a natural aromatase blocker (NAB) capable of blocking the aromatase enzyme from converting testosterone to estradiol. This generates consistently high levels of testosterone. This study aimed to determine the effect of blood clam shell powder (BCSP) as an NAB on the growth, pectoralis muscle performance, and testes of pelung cockerels.

**Materials and Methods:**

The study design was a completely randomized design, with 16 pelung cockerels aged 40–56 weeks divided into four treatment groups: T0 (control); T1 (BCSP [*A. granosa*] 0.9 mg/kg BW); T2 (zinc sulfate [ZnSO_4_] 0.9 mg/kg BW); and T3 (testosterone 3 mg/day). The animals were acclimatized for 7 days and then given dietary treatments for 56 days. The measurement of the comb, wattle, and chest circumference (CC) of pelung cockerels was performed on days 0, 14, 28, 42, and 56. At the end of the treatment, the pelung cockerels were sacrificed and the data of the pectoralis muscle weight (PMW), testis weight (TW), and area of the pectoralis muscle (APM) were measured. Samples of pectoralis muscle and testes were taken and fixed in 10% neutral buffer formalin for histology. The proliferating cell nuclear antigen (PCNA) was identified by immunohistochemical staining. To measure fascicle area (FA), myofiber area (MA), and enumerate, the fascicle myofibers (NM) histology preparations were stained with hematoxylin and eosin (H and E). Testicular preparations were stained with H and E to measure the diameter of the seminiferous tubules (DST) using ImageJ software.

**Results:**

The growth performance on day 56 showed significantly (p < 0.05) higher differences of CC in T1 compared to T2 and T0, in T1 and T3 compared to T0, and in T3 and T2 compared to T0. Pectoralis muscle results, that is, FA, NM, MA, and PCNA-positive cells, showed that cockerels on treatment T3 had significantly higher results than other treatments, T1 was significantly different from T2 and T0, and T2 was significantly different from T0. In addition, the TW and DST measurement of cockerels on treatment T3 were significantly reduced (p < 0.05) than the other treatment groups.

**Conclusion:**

The oral administration of BCSP in the role of a NAB at a dose of 0.9 mg/kg BW for 56 days improved the growth performance and pectoralis muscle, especially the CC, FA, NM, MA, and PCNA-positive cells parameters, but did not affect the PMW, APM, and testis of pelung cockerels. The administration of testosterone at 3 mg/day for 56 days contributed to the decrease in TW and DST, as well as atrophy of the seminiferous tubules of pelung cockerels.

## Introduction

Pelung cockerels are native to Cianjur, a district in West Java, Indonesia [1–3]. The animal has been settled under the 2918/kpts/OT.140/6/2011 decree issued by the Ministry of Agriculture as native Indonesian free-range chickens and is obliged to be protected and conserved [4]. The chicken is reported to be threatened due to a decrease in population size [1]. The pelung cockerels have a long, distinct melodious voice and are in high demand for crossbreeding with other local chickens [5]. In addition, pelung is a relatively fast-grow chicken with a large posture and strong appearance [5]. Male pelung cockerels produce good quality meat as shown by their high muscle mass and increased strength [6, 7]. The chest area or the pectoralis muscle has a low-fat content [8]. Improvement of pectoralis muscle is characterized by the increasing number of myofibers in one fascicle, myofiber area (MA), fascicle area (FA), and proliferation in the nucleus of muscle cells [9–12]. The increase in growth and the pectoralis muscle characteristics of pelung cockerels is triggered by the hormone testosterone [5, 7, 13–19]. The hormone testosterone in male animals is produced by the testes [5]. A common practice to increase testosterone levels is through parenteral synthetic testosterone [18]. The continuous administration of parenteral synthetic testosterone can lead to decreased testis weight (TW), seminiferous tubule damage, and infertility [5, 18, 20]. Therefore, a natural agent capable of acting as a natural aromatase blocker (NAB) is required, and blood clam shells may be an option [21].

Blood clams (*Anadara granosa*) are classified as bivalves. The shells are generally discarded after the meat is harvested which creates environmental challenges [22, 23]. According to Astuti *et al*. [24], the blood clam shells contain Zn, Mg, Fe, Ca, Na, and K, while the blood clam shell powder (BCSP) contains 30%–40% Ca, 1% P, and 3%–4% protein [25, 26]. The Zn content in the BCSP acts as a NAB which is able to increase testosterone hormone levels, antioxidants, growth performance, and modulate the immune system [24, 27–31]. The BCSP also contains proteins which are required to boost chicken growth, pectoralis muscle area, MA, and stimulate satellite cell proliferation to regenerate myofiber for muscle growth [32–36]. The BCSP used as a NAB at a dose of 0.036 mg/40 g body weight (BW) has been proven to increase TW at the 5^th^ week and serum testosterone levels in male layer breeders at the 4^th^ week post-treatment [18]. Astuti *et al*. [24] showed that 0.18 mg/200 g BW of BCSP supplemented for 50 days was able to increase testosterone levels and showed aromatase enzyme blocking action in the brain and testes of rats as evidenced by reduced CYP19 aromatase expression in immunohistochemical (IHC) staining. However, Yuneldi *et al*. [5] showed that *A. granosa* shell powder as a NAB increased crowing frequency, BW, and testosterone levels. The application of the NAB from BCSP has been proven to increase testosterone levels. Aromatase blocker is known to affect growth, pectoralis muscle performance, and testicular organs in animals. However, the use of the NAB from BCSP in male pelung cockerels has not been investigated.

Therefore, this study aimed to evaluate the effect of BCSP as a NAB on growth, pectoralis muscle performance, and testicular organs in pelung cockerels.

## Materials and Methods

### Ethical approval

All the research procedures have been approved by the Ethics Committee of Integrated Testing and Research, Universitas Gadjah Mada (Approval no. 00020/04/LPPT/V/2020).

### Study period and location

The study was conducted from September 2021 to March 2022 at the pelung cockerels farm in Bantul, Yogyakarta, Animal Development Structure Laboratory, Faculty of Biology and Physiology Laboratory, Faculty of Veterinary Medicine, Universitas Gadjah Mada, Indonesia.

### Preparation of the *A. granosa* BCSP

*Anadara granosa* BCSP was processed according to the procedure outlined by Yuneldi *et al*. [5]. After discarding the meat from the shells by boiling, the shells were cleaned and sundried for 1–2 days. Subsequently, the shells were boiled in NaOH solution (1.0 N) at 50°C for 3 h, rinsed under running water, and dried at 120°C for 6–8 h before being ground into a powder. The prepared BCSP was analyzed for its mineral content by inductively coupled plasma analysis. The clam shell of *A. granosa* contains Zn=61.55 mg/kg, Mg=1666.09 mg/kg, Fe=600.54 mg/kg, Ca = 41.4 mg/dL, Na=9262.98 mg/kg, and K=369.29 mg/kg [24].

### Experimental design and parameters

The study used 16 male pelung cockerels (sample size was small as the bird falls under the threatened species category) aged 40–56 weeks. The physiology performance of male pelung cockerels generally degrades from 40 to 56 weeks. To distinctively evaluate the effect of the NAB treatment, this specific age of the chicken was chosen in this study. The BW of the pelung cockerels was ±3 kg and they were randomly divided into four treatment groups: T0 (control); T1 (BCSP [*A. granosa*] at 0.9 mg/kg BW); T2 (zinc sulfate [ZnSO_4_] at 0.9 mg/kg BW); and T3 (testosterone at 3 mg/day). Each treatment group contained four pelung cockerels (n = 4). The BCSP and ZnSO_4_ were orally administered through gavage [5, 18, 24, 37] and testosterone was administered subcutaneously [18, 31]. The treatment was administered to the cockerels for 56 days. Drinking water and commercial breeder feed were provided *ad libitum* [38]. Before treatment, the pelung cockerels were allowed to acclimatize for 7 days. Measurements taken in growth performance were comb length and height, wattle area, and chest circumference (CC). The measurements for pectoralis muscle observation were pectoralis muscle weight (PMW), area of the pectoralis muscle (APM), FA, fascicle myofibers (NM), MA, and proliferating cell nuclear antigen (PCNA)-positive cells. The measurements for testes were TW and diameter of the seminiferous tubules (DST). Measurements of CC, comb length and height, and wattle area were collected fortnightly on days 0, 14, 28, 42, and 56.

### Comb morphometry, wattle, and CC

Comb and wattle measurements were performed using a pair of calipers, while the CC was taken using a measuring tape [14, 39, 40].

### Euthanasia and organ preparation

On the 56^th^ day, the cockerels were sacrificed using the halal method. The chest muscles and testicles were collected and washed with 0.9% NaCl [41, 42]. The testes and left pectoralis muscle were weighed and right muscle was measured using ImageJ software [32]. Subsequently, they were processed for histological examination [18, 32].

### Hematoxylin and eosin staining (H & E)

For histological analysis, the pectoralis muscle and testes were fixed in 10% neutral buffer formalin (NBF) solution for 18–24 h and stained with H and E (Merck, Darmstadt, Germany) [24, 32, 43]. Furthermore, the slides were observed under a microscope (Leica DM 750, Germany) with magnifications of 10 × 10 and 40 × 10. The area of myofibers and fasciculus were measured, and the number of myofibers in one fascicle enumerated. Testicular histological slides were observed and the DST measured. These measurements were obtained using ImageJ software [32].

### Immunohistochemical staining

The pectoralis muscle samples were fixed in 10% NBF solution for 18–24 h; they were, then cut into 5 mm thick sections and placed onto a poly-l-lysine coated slide. The slides were stained with the immunohistochemistry method using EnVision + system – HRP (DAB) with anti-PCNA primary antibody (PC10) ab29 (Abcam, USA) to detect cell proliferation. The secondary antibody used was polyclonal goat anti-mouse immunoglobulins (IgG) (Dako, Glostrup, Denmark) and color development was enhanced using diaminobenzidine (Dako). The preparations were mounted using Entellan (Merck) and covered with a 24 × 60 mm glass coverslip. Following indirect IHC staining, the slides were then observed for the muscle cell nuclei. Proliferating cell nuclear antigen-positive cells are indicated with brown nuclei in the pectoralis muscle cells (modification of 11, 12). The percentage of PCNA-positive nuclei was calculated by counting the number of PCNA-positive nuclei divided by number of nuclei observed in the myofibers in one fascicle multiplied by 100% (modification of 11, 12). Pectoralis muscle histological slides were examined, and the total myofiber in one fascicle was counted, and the area of fasciculus and myofiber was determined. The measurement of those parameters was obtained using ImageJ software [36, 44].

### Statistical analysis

All parameters were statistically analyzed with one-way analysis of variance using a statistical package for the social sciences software v.26.0 (IBM, NY, USA) with a 95% confidence level (α = 0.05). The analysis was confirmed with Duncan’s test [45].

## Results

### Comb and wattle

The comb length, height, and wattle area of the pelung cockerels at day 56 did not show significant differences (p > 0.05) between the treatments (Table-1).

**Table-1:** The average comb length, comb height, and wattle area of male pelung cockerels.

T	Average ± SD comb length (cm), day				

	0	14	28	42	56
T0	11.57 ± 2.27	11.80 ± 2.17	12.13 ± 2.29	12.71 ± 1.84	12.74 ± 1.85
T1	12.95 ± 2.21	13.09 ± 2.23	13.49 ± 2.51	13.60 ± 2.56	13.77 ± 2.54
T2	11.72 ± 0.66	11.82 ± 0.62	11.90 ± 0.61	12.42 ± 0.49	12.82 ± 0.92
T3	12.20 ± 3.54	13.50 ± 2.73	13.87 ± 2.82	14.05 ± 2.76	14.92 ± 1.55

**Average ± SD comb height (cm)**					

T0	6.00 ± 1.29	6.27 ± 1.17	6.55 ± 1.10	6.85 ± 1.43	7.02 ± 1.62
T1	6.02 ± 1.36	6.52 ± 1.44	6.70 ± 1.40	7.00 ± 1.41	7.45 ± 1.52
T2	6.00 ± 0.41	6.32 ± 0.34	6.67 ± 0.56	7.00 ± 0.73	7.20 ± 1.00
T3	7.20 ± 2.39	7.75 ± 1.65	8.05 ± 1.44	8.87 ± 1.65	9.45 ± 1.22

**Average ± SD wattle area (cm^2^)**					

T0	20.60 ± 4.67	20.60 ± 4.67	28.75 ± 7.28	30.00 ± 6.33	31.25 ± 8.79
T1	38.61 ± 22.10	41.17 ± 23.34	44.02 ± 23.31	44.85 ± 25.41	45.26 ± 26.48
T2	21.90 ± 2.29	22.40 ± 1.84	28.90 ± 5.44	30.53 ± 6.61	33.00 ± 9.68
T3	47.07 ± 27.24	50.12 ± 24.73	57.12 ± 22.81	60.78 ± 21.11	63.95 ± 18.97

No superscript indicates no significant difference. T0=Control, T1=Blood clam shell powder (*A. granosa*) 0.9 mg/kg BW, T2=ZnSO_4_ 0.9 mg/kg BW, T3=Testosterone 3 mg/day, SD=Standard deviation, T=Treatment

### Chest circumference

The CC at day 56 showed that T1 was significantly higher (p < 0.05) than those of T0 and T2 (Table-2). T1, T2, and T3 were significantly higher than T0 (Table-2).

**Table-2:** The average CC of male pelung cockerels.

T	Average ± SD CC (cm), day				

	0	14	28	42	56
T0	38.37 ± 2.68	38.50 ± 2.88^b^	39.25 ± 3.30^b^	39.50 ± 2.88^c^	41.25 ± 1.70^c^
T1	40.37 ± 1.79^i^	43.50 ± 1.29^a,h^	44.62 ± 1.10^a,gh^	44.75 ± 0.95^a,gh^	45.62 ± 0.47^a,g^
T2	38.00 ± 2.64^i^	39.62 ± 1.10^b,hi^	39.62 ± 1.10^b,hi^	41.50 ± 2.08^bc,gh^	43.25 ± 1.70^b,g^
T3	37.37 ± 1.25^h^	39.12 ± 2.25^b,h^	42.75 ± 0.95^a,g^	43.50 ± 1.29^ab,g^	44.00 ± 0.81^ab,g^

^a-c^Mean with different superscripts within the same column are significantly different (p < 0.05). ^g-i^Mean with different superscripts within the same row are significantly different (p < 0.05). No superscript indicates no significant difference. T0=Control, T1=Blood clam shell powder (*A. granosa*) 0.9 mg/kg BW, T2=ZnSO_4_ 0.9 mg/kg BW, T3=Testosterone 3 mg/day, SD=Standard deviation, T=Treatment, CC=Chest circumference

### Pectoralis muscle weight, pectoralis muscle area, FA, number of myofiber in one fascicle, MA, and PCNA-positive cells

**Figure-1:**
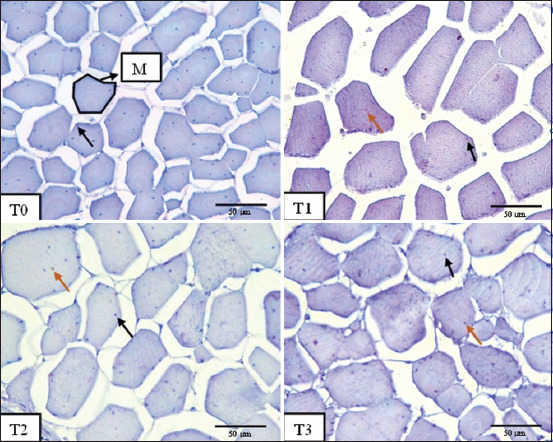
Histology of the pectoralis muscle of male pelung cockerels aged 40–56 weeks stained with immunohistochemical staining after 56 days of treatment. Description: T0=Control, T1=0.9 mg/kg body weight of blood clam shell powder (*A. granosa*), T2=0.9 mg/kg body weight ZnSO_4_, T3=3 mg/day testosterone. Magnification: 40 × 10. M=Myofiber. Immunohistochemical staining of anti-proliferating cell nuclear antigen (PCNA). “→”: PCNA=Positive muscle cells marked with brown nuclei and “→”: Nucleus in muscle cells, T=Treatment.

The weight and APM showed no significant difference between all the treatments (p > 0.05) (Table-3). However, the FA, NM, MA, and PCNA-positive cells showed that the T3 pelung cockerels had significantly higher values (p < 0.05) compared to the other treatments, T1 was significantly different (p < 0.05) from T2 and T0, and T2 was significantly different (p < 0.05) from T0 (Table-3 and Figure-1).

**Table-3:** The average of PMW, pectoralis muscle area, FA, number of myofibers in one fascicle, MA, and PCNA-positive cells in male pelung cockerels aged 40–56 weeks after 56 days of treatment.

T	Average ± SD, parameters					

	PMW (g)	Pectoralis muscle area (cm^2^)	FA (mm^2^)	Number of myofibers in one fascicle	MA (mm^2^)	PCNA- positive cells (%)
T0	164.35 ± 37.05	119.86 ± 21.72	212.38×10^-3^ ± 12.42×10^-3d^	103.91 ± 3.20^d^	1.948×10^-3^ ± 0.012×10^-3d^	0.83 ± 0.43^d^
T1	179.67 ± 5.38	137.50 ± 13.48	329.88×10^-3^ ± 7.05×10^-3b^	114.16 ± 5.35^b^	2.646×10^-3^ ± 0.028×10^-3b^	5.41 ± 0.41^b^
T2	172.66 ± 16.81	126.94 ± 21.13	281.94×10^-3^ ± 13.50×10^-3c^	109.16 ± 2.36^c^	2.180×10^-3^ ± 0.028×10^-3c^	3.75 ± 0.31^c^
T3	173.02 ± 17.54	127.27 ± 19.76	397.07×10^-3^ ± 8.11×10^-3a^	128.91 ± 4.29^a^	3.612×10^-3^ ± 0.027×10^-3a^	10.66 ± 0.72^a^

^a-d^Mean with different superscripts within the same column are significantly different (p < 0.05). No superscript indicates no significant difference. T0=Control, T1=Blood clam shell powder (*A. granosa*) 0.9 mg/kg BW, T2=ZnSO_4_ 0.9 mg/kg BW, T3=Testosterone 3 mg/day, SD=Standard deviation, T=Treatment, PMW=Pectoralis muscle weight, FA=Fascicle area, MA=Myofiber area, PCNA=Proliferating cell nuclear antigen

### Testis weight (TW) and seminiferous tubule diameter

**Figure-2:**
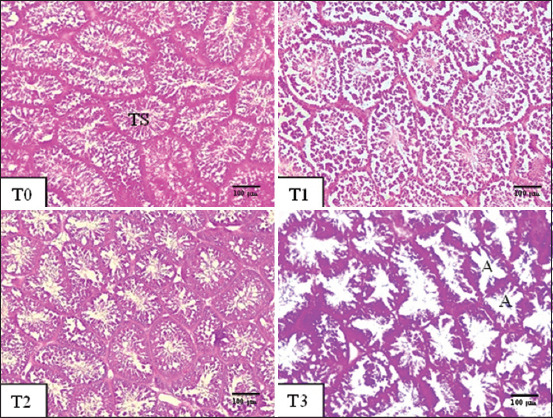
Testicular histology of male pelung cockerels aged 40–56 weeks after 56 days of treatment. Description: T0=Control, T1=0.9 mg/kg body weight of blood clam shell powder (BCSP) (*A. granosa*), T2=0.9 mg/kg body weight ZnSO_4_, T3=3 mg/day testosterone. Magnification: 10 × 10. Hematoxylin-eosin staining. TS=Seminiferous tubules, A=Atrophy, T=Treatment.

The statistical analysis result of DST (Figure-2) and TW showed that the T3 pelung cockerels were significantly reduced (p < 0.05) compared to the other groups (Table-4).

**Table-4:** The average of testis weight and seminiferous tubule diameter of aged male pelung cockerels 40–56 weeks after treatment for 56 days.

T	Average ± SD, parameters	

	TW (g)	Diameter of seminiferous tubule (μm^2^)
T0	29.00 ± 3.55^a^	113.01 ± 7.90^a^
T1	35.25 ± 5.50^a^	119.39 ± 8.40^a^
T2	28.83 ± 4.60^a^	111.93 ± 8.35^a^
T3	17.57 ± 2.79^b^	77.97 ± 9.96^b^

^a,b^Mean with different superscripts within the same column are significantly different (p < 0.05). T0=Control, T1=Blood clam shell powder (BCSP) (*A. granosa*) 0.9 mg/kg BW, T2=ZnSO_4_ 0.9 mg/kg BW, T3=Testosterone 3 mg/day, SD=Standard deviation, T=Treatment, TW: Testis weight

## Discussion

### Comb and wattle 56 days post-treatment

The length and height of the comb, and the wattle area were not significantly different as chickens used in this study were adult pelung cockerels. The growth period of the comb and wattles was slow and some had reached full growth size. These results are in agreement with Astuti *et al*. [17], that demonstrated that the administration of *Anadara nodifera* shell powder as a NAB at doses of 3.3 and 6.6 g/day, ZnSO_4_ 0.45 mg/kg BW, and testosterone 0.1 mL/day for 35 days did not increase the size of the comb and wattle in adult Bangkok cockerels. This was also shown by Yuneldi *et al*. [18], where the administration of BCSP as a NAB at a dose of 0.036 mg/40 g BW and ZnSO_4_ 0.018 mg/40 g BW for 35 days could not increase the length and height of the day old chick (DOC) comb of male layers.

### Chest circumference, PMW, pectoralis muscle area, FA, number of myofiber in one fascicle, MA, and PCNA-positive cells 56 days post-treatment

There was no previous information on the administration of BCSP (T1) on CC, PMW, APM, FA, NM, MA, and PCNA-positive cells in pelung cockerels. The results showed that the administration of BSCP was able to increase the CC in pelung cockerels. This may be due to the Zn contained in the BCSP as a NAB, which contributes to the biological activity and metabolic action in the digestion of pelung cockerels. This is in agreement with Sandoval *et al*. [46] and Bartlett and Smith [47] that Zn can improve the growth and health performance of broilers. In addition, Kakhki *et al*. [48] reported that supplementation with Zn at a dose of 120 mg/kg affected the growth performance of broiler breast muscles. Besides acting as a NAB, Zn also acts as an antioxidant [49]. Reed *et al*. [50] reported that Zn positively contributes to the digestive system in chickens. It is estimated that the mineral composition and physiological function of the BSCP are involved in the metabolic system which has an impact on increasing the pelung cockerels CC. Another possibility is the added protein content in the BCSP. The previous research have been reported that the protein content in chicken feed has a considerable influence on the growth of chickens and increases the efficiency of their digestive system [32, 34, 35].

This study showed that the treatments in groups T1, T2, and T3 were effective in improving the FA, NM, MA, and PCNA-positive cells of pelung cockerels compared to T0. Group T3 was significantly higher than the other groups. Group T1 was significantly higher than groups T2 and T0. T2 group was higher than T0. The Zn contained in BCSP can increase testosterone which further affected the FA, NM, MA, and PCNA-positive cells. These results are in accordance with Yuneldi *et al*. [5] and Astuti *et al*. [19, 24], who showed that *A. granosa* shell powder at 0.9 mg/kg BW, 0.3 mg/30 g BW, and 0.18 mg/200 g BW was capable of increasing the testosterone levels in pelung cockerels, canaries, and rats. According to Li *et al*. [7], the elevation of testosterone can significantly increase the FA, NM, and MA, and according to Taylor *et al*. [51], Zn can increase the proliferation of muscle cells nuclei. Bonaventura *et al*. [52] demonstrated that Zn plays an important role in cellular proliferation and differentiation. The possible mechanism by which BCSP as a NAB can increase testosterone levels is that in the endoplasmic reticulum, Zn^2+^ as a second messenger activates CK2, which adds a phosphate group (phosphorylation) to aromatase [53–55]. High intracellular phosphorylation can inhibit aromatase enzyme activity which inhibits the conversion of testosterone to estradiol [5, 24, 55, 56]. This causes an elevation of the hormone testosterone [19, 51, 55–57]. This is supported by Yuneldi *et al*. [18], who demonstrated that BCSP at 0.036 mg/40 g BW as a NAB increased testosterone levels in male layer chicks. High testosterone levels and balanced nutritional intake (adequate protein) can affect muscle mass and strength [58]. High testosterone from the mechanism of Zn as a NAB increases the number of active satellite cells in the skeletal muscle and transcription processes occur in the myonucleus [6].

There are two pathways of testosterone action in skeletal muscle. First, the androgen receptor located in the myonucleus binds to the hormone testosterone. This process increases the production of insulin-like growth factor 1, which stimulates increased protein synthesis and suppresses protein catabolism [59]. Second, the activated satellite cells express myogenic regulatory factors during skeletal muscle development, initiate proliferation and differentiation processes, and fuse to form new myotubes [6]. Furthermore, it undergoes myofiber maturation and increases the number of new myonuclei [59]. The above explanation demonstrates the process that occurs during muscle hyperplasia and hypertrophy. A similar concept has been affirmed by the work of Hughes *et al*. [60]. High testosterone accompanied by adequate nutrition can affect muscle mass and strength [58]. In addition, the myostatin gene or growth and differentiation factor 8 belong to the transforming growth factor-beta superfamily, serve as skeletal muscle growth regulation and differentiation factors [61, 62].

Besides functioning as a NAB, Zn can also act as an antioxidant. According to Prasad and Kucuk [63] and Rouhalamini *et al*. [64], Zn can perform an antioxidant role that can reduce oxidative stress in chickens due to heat stress. Heat stress in chickens can cause muscle protein degeneration and a decrease in size and MA, which ultimately reduces muscle mass [31]. According to Chand *et al*. [65], Khan *et al*. [66], and Naz *et al*. [67], the function of Zn in chickens is to overcome heat stress. Zinc administration as an antioxidant can reduce oxidative stress and therefore improves meat quality [64] by increasing MA and FA [31]. Increased FA may be associated with increased NM and MA. According to Shah *et al*. [68], an increase in FA is associated with an increase in the amount of myofibers and MA because the fasciculus consists of a collection of myofibers, and the increased fascicle cross-sectional area is associated with the increased diameter and cross-sectional area of myofibers in the pectoralis muscle of chickens [31]. Muscle growth can be observed from FA, NM, and MA measurements. Muscle growth after birth is noted by the increased size of myofibers or number of myofibers [69]. The increase of muscle mass during postnatal growth occurs due to muscle hypertrophy and hyperplasia [70–72]. Another possible factor affecting the parameters of FA, NM, and MA other than the minerals, is the protein content of the BCSP. According to Saragih *et al*. [36], the MA of broiler and pelung chickens that are fed a high protein feed on days 7 and 14 was increased compared to the broiler and pelung chickens fed a low protein feed. These studies results are consistent with those of Saragih *et al*. [32] and Paunesku *et al*. [33] in that providing sufficient protein in chicken feed is important for pectoralis muscle growth and protein is known to stimulate satellite cell proliferation in regenerating myofibers for improved muscle growth.

The results of FA, NM, MA, and cells that were positive for PCNA measurements of pelung cockerels treated with testosterone (T3) showed a significant increase compared to the other treatments. According to Li *et al*. [7], the administration of exogenous testosterone significantly increases the NM and MA of the pectoralis muscle of chickens and supported muscle growth by increasing the proliferation of chicken muscle cells. According to Josiak *et al*. [6], it was suggested that the active satellite cells proliferate and differentiate to form new myotubes. Furthermore, myofiber maturation occurs and increases the number of new myonuclei. This supports the process of muscle hyperplasia and hypertrophy [6]. Other studies have affirmed this concept [7, 73–76].

### Testicular weight and DST 56 days post-treatment

The TW and DST of the pelung cockerels on treatments T0, T1, and T2 did not show any significant differences. The effect of BCSP on the DST has not been previously reported. According to Yuneldi *et al*. [18], using BCSP as a NAB at a dose of 0.036 mg/40 g BW and treatment with ZnSO_4_ 0.018 mg/40 g BW had no effect on the TW of DOC male layers. A similar result with *A. nodifera* shell powder as a NAB was obtained at a dose of 3.3 g/day and ZnSO_4_ 0.45 mg/kg for 35 days for Bangkok chickens [17]. According to Adelakun *et al*. [77], the ZnSO_4_ treatment in rats for 56 days did not affect the DST compared to the controls. In addition, ZnSO_4_ treatment to mice for 30 days did not affect the DST compared to the controls [78]. Thus, in accordance with the above-mentioned studies, BCSP and ZnSO_4_ treatment did not affect the TW and DST.

The results of this study on the TW and DST showed that pelung cockerels treated with testosterone (T3) experienced a significant decrease compared to other treatments. According to Yuneldi *et al*. [18], the continuous administration of 3 mg/day of testosterone for 35 days in male layer chicken significantly reduces the TW. Similar results of continuous administration of testosterone at 4 mg/head/day to Bangkok chickens for 35 days led to a decrease in TW [17]. The application of exogenous testosterone suppresses the secretion of endogenous testosterone through a negative feedback mechanism in the hypothalamic-pituitary-anterior axis, luteinizing hormone (LH), and follicle-stimulating hormone (FSH) [79]. Disturbances in the secretion of endogenous testosterone, LH, and FSH hormones can lead to decreased TW or testicular atrophy in rats [80]. According to Amer and Selim [20], administering exogenous testosterone at a dose of 5 mg/kg for 6 days/week for 4 weeks caused seminiferous tubule atrophy and reduced the DST in rats. Testosterone propionate at a dose of 3 mg/100 g BW for 60 days also reduced the DST in rats [81], and the increased serum testosterone levels caused by the administration of exogenous testosterone inhibited the hypothalamic-pituitary-testicular axis in rats [20]. According to Yama *et al*. [82], the decrease in DST could be caused by the inhibition of LH secretion by the anterior pituitary, which functions to stimulate the growth and number of Leydig cells in mice. Furthermore, when LH is inhibited that the Leydig cells are inactivated and do not produce testosterone, resulting in a decreased testosterone level in the testes [83]. This lack of testosterone and FSH levels is predicted to cause seminiferous tubular atrophy in rats [20]. When the Leydig cells are not stimulated by the hypothalamus and pituitary gland to synthesize testosterone for a relatively extended period of time, testicular degeneration occurs and inhibition of FSH and LH secretion causes a reduction in the DST [83].

## Conclusion

The oral administration of BCSP as a NAB at a dose of 0.9 mg/kg BW (T1) for 56 days improved the growth performance and pectoralis muscle, especially the CC, FA, NM, MA, and PCNA-positive cells, but did not affect the PMW, APM, and testes of the pelung cockerels. However, administering testosterone at 3 mg/day (T3) for 56 days caused the side effect of reduced TW and DST and induced atrophy of the seminiferous tubules of pelung cockerels.

## Authors’ Contributions

PA, CMA, HTSS, and RFY: Planned, designed, and contributed to the experiment. PA, CMA, HTSS, RFY, SS, ARA: Recorded and analyzed the samples and drafted and edited the manuscript. All authors have read, reviewed, and approved the final manuscript.

## References

[1] Asmara I.Y, Garnida D, Partasasmita R (2020). Crowing characteristics of Pelung chickens at different age and body weight. Biodivers. J. Biol. Divers.

[2] Asmara I.Y, Garnida D, Partasasmita R (2020). Short communication:Duration and volume of crowing of Pelung chickens of West Java, Indonesia. Biodivers. J. Biol. Divers.

[3] Daryono B.S, Mushlih M, Perdamaian A.B.I (2021). Crowing sound and inbreeding coefficient analysis of Pelung chicken (*Gallus gallus domesticus*). Biodivers. J. Biol. Divers.

[4] Ministry of Agriculture. Keputusan Menteri Pertanian Nomor 2918/kpts/OT.140/6/2011 Tentang Penetapan Rumpun Ayam Pelung (2011). Ministry of Agriculture of the Republic of Indonesia, Jakarta, Indonesia.

[5] Yuneldi R.F, Astuti P, Saragih H.T.S, Airin C.M (2021). *Anadara granosa* shell powder improves the metabolism, testosterone level, and sound frequency of Pelung chickens. Vet. World.

[6] Josiak K, Jankowska E.A, Piepoli M.F, Banasiak W, Ponikowski P (2014). Skeletal myopathy in patients with chronic heart failure:Significance of anabolic-androgenic hormones. J. Cachexia Sarcopenia Muscle.

[7] Li D, Wang Q, Shi K, Lu Y, Yu D, Shi X, Du W, Yu M (2020). Testosterone promotes the proliferation of chicken embryonic myoblasts via androgen receptor mediated Pi3K/Akt signaling pathway. Int. J. Mol. Sci.

[8] Velleman S.G (2020). Pectoralis major (breast) muscle extracellular matrix fibrillar collagen modifications associated with the wooden breast fibrotic myopathy in broilers. Front. Physiol.

[9] Lembayu R.P.L, Armandu A.C, Saragih H.T (2022). Histological structure of pectoralis thoracicus, small intestine, and growth performance of broiler chicken after supplementation of peanut hulls (*Arachis hypogaea* L.) *Indones*. J. Anim. Sci.

[10] Saragih H.T.S.S, Daryono B.S (2012). Effect of high-protein diet on body weight and pectoralis thoracicus muscle performance on Pelung and broiler chicken (*Gallus gallus domesticus*). Anim. Prod.

[11] Puspita U.E, Utomo R.T, Perdamaian A.B.I, Lesmana I, Arijuddin H, Erwanto Y, Daryono B.S, Saragih H.T.S.G (2017). Effect of varying levels of protein and energy in pre-stater feeds on pectoralis muscle development of kampung super chicks (*Gallus gallus gallus*). Asian J. Anim. Vet. Adv.

[12] Zielinska M.K, Sawosz E, Chwalibog A, Ostaszewska T, Kamaszewski M, Grodzik M, Skomiał J (2010). Nano-nutrition of chicken embryos. Effect of gold and taurine nanoparticles on muscle development. J. Anim. Feed Sci.

[13] Kilic M (2007). Effect of fatiguing bicycle exercise on thyroid hormone and testosterone levels in sedentary males supplemented with oral zinc. Neuro. Endocrinol. Lett.

[14] Santoso M.S, Tana S, Mardiati S.M (2010). Effect of virgin coconut oil (VCO) addition on cockscomb development and chicken testicular weight (*Gallus* spp.). Bull. Anat. Physiol.

[15] Sato K, Iemitsu M, Matsutani K, Kurihara T, Hamaoka T, Fujita S (2014). Resistance training restores muscle sex steroid hormone steroidogenesis in older men. FASEB J.

[16] Alward B.A, Balthazart J, Ball G.F (2017). Dissociable effects on birdsong of androgen signaling in cortex-like brain regions of canaries. J. Neurosci.

[17] Astuti P, Airin C.M, Hana R.R.A, Yuneldi R.F, Sarmin S (2021). The effect of natural aromatase blockers on the testicle weight, size of wattle and histopathological of testis in Bangkok. Bio Web Conf, EDP Sciences.

[18] Yuneldi R.F, Airin C.M, Saragih H.T.S, Astuti P (2021). Application of natural aromatase blocker towards the level of testosterone in rooster layer [*Gallus gallus gallus* (Linn., 1758)]. In:Key Engineering Materials.

[19] Astuti P, Putra M.N.P, Shiddiq M.F.A, Yuneldi R.F, Airin C.M, Sarmin S (2022). The potency of *Anadara nodifera* shell as natural testosterone booster for male canary (*Seriunus canaria*). HAYATI J. Biosci.

[20] Amer M.G, Selim A.O (2011). Histological changes induced by testosterone abuse in the testis and the skeletal muscle of adult male albino rats. Egypt. J. Histol.

[21] Astuti P, Airin C.M, Nururrozi A, Harimurti S (2018). Oyster Shell Powder as Alternatives Macromineral for Synthetic Testosterone. In:Proceedings of the 20^th^ FAVA Congress and 15^th^ KIVNAS PDH, Bali, Indonesia.

[22] Nirmalasari R (2017). The effect of feeding blood cockle *Anadara granosa* L. on the spermatozoa density of *Mus musculus* L. BIOMA J. Biol. Makassar.

[23] Nguyen T.A, Nhan C.H, Le M.V, Huynh P.H.K, Phung T.K, Tran A.V (2021). Fixed bed column studies for the adsorption of cadmium onto cockle shell (*Anadara granosa*) powder. Chem. Eng. Trans. AIDIC.

[24] Astuti P, Airin C.M, Sarmin S, Nururrozi A, Harimurti S (2019). Effect of shell as natural testosterone boosters in Sprague Dawley rats. Vet. World.

[25] Kurniasih D, Rahmat M.B, Handoko C.R, Arfianto A.Z (2017). Making animal feed from clamshell waste in Bulak Kenjeran village, Surabaya. Lemonmen. PPNS Master.

[26] Mahary A (2017). The application of blood cockle (*Anadara granosa*) shell as calcium source for catfish (*Clarias batrachus* sp) feed. Aquat. Sci. J.

[27] Liu Z.H, Lu L, Li S.F, Zhang L.Y, Xi L, Zhang K.Y, Luo X.G (2011). Effects of supplemental zinc source and level on growth performance, carcass traits, and meat quality of broilers. Poult. Sci.

[28] El-Husseiny O.M, Hashish S.M, Ali R.A, Arafa S.A, El-Samee L.D.A, Olemy A.A (2012). Effects of feeding organic zinc, manganese and copper on broiler growth, carcass characteristics, bone quality and mineral content in bone, liver and excreta. Int. J. Poult. Sci.

[29] Akhuemokhan I.K, Eregie A, Fasanmade O.A (2013). Diabetes prevention and management:The role of trace minerals. Afr. J. Diabetes Med.

[30] Chu Q, Chi Z.H, Zhang X, Liang D, Wang X, Zhao Y, Zhang L, Zhang P (2016). A potential role for zinc transporter 7 in testosterone synthesis in mouse Leydig tumor cells. Int. J. Mol. Med.

[31] Shah M, Zaneb H, Masood S, Khan R.U, Din S, Khan I, Shakirullah S, ur-Rehman H, Tariq A (2019). Ameliorative effect of zinc and multistrain probiotic on muscle and bone characteristics in broiler reared under cyclic heat stress. Pak. J. Zool.

[32] Saragih H.T, Muhamad A.A.K, Alfianto A, Viniwidihastuti F, Untari L.F, Lesmana I, Widyatmoko H, Rohmah Z (2019). Effects of *Spirogyra jaoensis* as a dietary supplement on growth, pectoralis muscle performance, and small intestine morphology of broiler chickens. Vet. World.

[33] Paunesku T, Mittal S, Protić M, Oryhon J, Korolev S.V, Joachimiak A, Woloschak G.E (2001). Proliferating cell nuclear antigen (PCNA):Ringmaster of the genome. Int. J. Radiat. Biol.

[34] Ravindran V (2013). Poultry feed availability and nutrition in developing countries. In:Poultry Development Review. Food and Agriculture Organization of the United Nations, Rome.

[35] Beski S.S.M, Swick R.A, Iji P.A (2015). Specialized protein products in broiler chicken nutrition:A review. Anim. Nutr.

[36] Saragih H.T.S.G, Roosdianto I, Daryono B.S (2017). Pectoralis thoracicus muscle performance of hybrid chicken (F1) derived from crossbreed between broiler and pelung (*Gallus gallus gallus*). J. Kedokteran Hewan.

[37] Astuti P, Airin C.M, Nurrurozi A, Aidi R, Hana A, Hadi S, Harimurti H (2020). Potential natural aromatase blockers on enhance the frequency and sound quality of male canaries. E3S Web *Conf*. EDP Sciences.

[38] Elwahesh R.M, Ben-Elhaj K.M, Draid M.M (2016). Relationship between body weight performance and plasma thyroid hormones in broiler hens. Int. J. Med. Res. Prof.

[39] Abdel-Lattif F.H (2019). The linear association between live body weight and some body measurements in some chicken strains. Plant Arch.

[40] Sitanggang E.N, Hasnudi H, Hamdan H (2016). Diversity of qualitative trait and morphometrics between Kampung, Katai Birma, Bagon, and Magon chicken in Medan. J. Anim. Husb.

[41] Setiawan H, Jingga M.E, Saragih H.T (2018). The effect of cashew leaf extract on small intestine morphology and growth performance of Jawa Super chicken. Vet. World.

[42] Albab L.U, Claudya T.I, Oktafianti R, Salsabila N, Putri R.D, Saragih H.T.S.S (2022). Growth performance, morphometric of the small intestine, lymphoid organ, and ovary of laying hens supplemented with Dates (*Phoenix dactylifera* L.) extract in drinking water. Vet. World.

[43] Yuneldi R.F, Saraswati T.R, Yuniwarti E.Y.W (2021). The histomorphometry of liver and kidney of hyperglycemic albino rats after treatment with *Tithonia diversifolia* leaf extract. Biosaintifika J. Biol. Biol. Educ.

[44] Velleman S.G, Coy C.S, McFarland D.C (2007). Effect of syndecan-1, syndecan-4, and glypican-1 on turkey muscle satellite cell proliferation, differentiation, and responsiveness to fibroblast growth factor 2. Poult. Sci.

[45] Yuneldi R.F, Airin C.M, Saragih H.T.S, Astuti P (2022). The effect of natural aromatase blocker on the growth comb and body weight of layer chicken. Bio Web Conf, EDP Sciences.

[46] Sandoval M, Henry P.R, Littell R.C, Miles R.D, Butcher G.D, Ammerman C.B (1999). Effect of dietary zinc source and method of oral administration on performance and tissue trace mineral concentration of broiler chicks. J. Anim. Sci.

[47] Bartlett J.R, Smith M.O (2003). Effects of different levels of zinc on the performance and immunocompetence of broilers under heat stress. Poult. Sci.

[48] Kakhki R.A.M, Bakhshalinejad R, Hassanabadi A, Ferket P (2017). Effects of dietary organic zinc and a-tocopheryl acetate supplements on growth performance, meat quality, tissues minerals, and a-tocopherol deposition in broiler chickens. Poult. Sci.

[49] Nurjanah N, Zulhamsyah Z, Kustiyariyah K (2005). Mineral content and proximate of clamshell blood (*Anadara granosa*) taken from Boalemo regency, Gorontalo. Bull. Fisheries Prod. Technol.

[50] Reed S, Neuman H, Moscovich S, Glahn R.P, Koren O, Tako E (2015). Chronic zinc deficiency alters chick gut microbiota composition and function. Nutrient.

[51] Taylor K.M, Kille P, Hogstrand C (2012). Protein kinase CK2 opens the gate for zinc signaling. Cell Cycle.

[52] Bonaventura P, Benedetti G, Albarède F, Miossec P (2015). Zinc and its role in immunity and inflammation. Autoimmun. Rev.

[53] Kambe T, Tsuji T, Hashimoto A, Itsumura N (2015). The physiological, biochemical, and molecular roles of zinc transporters in zinc homeostasis and metabolism. Physiol. Rev.

[54] Kambe T, Taylor K.M, Fu D (2021). Zinc transporters and their functional integration in mammalian cells. J. Biol. Chem.

[55] Charlier T.D, Cornil C.A, Patte-Mensah C, Meyer L, Mensah-Nyagan A.G, Balthazart J (2015). Local modulation of steroid action:Rapid control of enzymatic activity. Front. Neurosci.

[56] Zhang X, Guan T, Yang B, Chi Z, Wang Z.Y, Gu H.F (2018). A novel role for zinc transporter 8 in the facilitation of zinc accumulation and regulation of testosterone synthesis in Leydig cells of human and mouse testicles. Metabolism.

[57] Santos H.O, Teixeira F.J (2020). Use of medicinal doses of zinc as a safe and efficient coadjutant in the treatment of male hypogonadism. Aging Male.

[58] Fu S, Lin X, Yin L, Wang X (2021). Androgen receptor regulates the proliferation of myoblasts under appropriate or excessive stretch through IGF-1 receptor mediated P38 and ERK1/2 pathways. Nutr. Metab. (Lond.

[59] Rossetti M.L, Steiner J.L, Gordon B.S (2017). Androgen-mediated regulation of skeletal muscle protein balance. Mol. Cell. Endocrinol.

[60] Hughes D.C, Stewart C.E, Sculthorpe N, Dugdale H.F, Yousefian F, Lewis M.P, Sharples A. P (2016). Testosterone enables growth and hypertrophy in fusion impaired myoblasts that display myotube atrophy:Deciphering the role of androgen and IGF-I receptors. Biogerontology.

[61] Sharma M, McFarlane C, Kambadur R, Kukreti H, Bonala S, Srinivasan S (2015). Myostatin:Expanding horizons. IUBMB Life.

[62] Tanjung A, Saragih H.T.S.S, Soenarwan H.P, Widianto S, Mahardhika I.W.S, Daryono B.S (2019). Polymorphism of myostatin gene and its association with body weight traits in a hybrid of GAMA chicken (*Gallus gallus domesticus* Linn 1758). Biodivers. J. Biol. Divers.

[63] Prasad A.S, Kucuk O (2002). Zinc in cancer prevention. Cancer Metastasis Rev.

[64] Rouhalamini S.M, Salarmoini M, Asadi-Karam G (2014). Effect of zinc sulfate and organic chromium supplementation on the performance, meat quality and immune response of Japanese quails under heat stress conditions. Poult. Sci. J.

[65] Chand N, Naz S, Khan A, Khan S, Khan R.U (2014). Performance traits and immune response of broiler chicks treated with zinc and ascorbic acid supplementation during cyclic heat stress. Int. J. Biometeorol.

[66] Khan R.U, Nikousefat Z, Javdani M, Tufarelli V, Laudadio V (2011). Zinc-induced molting:Production and physiology. Worlds Poult. Sci. J.

[67] Naz S, Idris M, Khalique M.A, Alhidary I.A, Abdelrahman M.M, Khan R.U, Chand N, Farooq U, Ahmad S (2016). The activity and use of zinc in poultry diets. Worlds Poult. Sci. J.

[68] Shah M, Zaneb H, Masood S, Qureshi A.S, Ullah H.A, Sikandar A, Din S, Ahmad I, Khan M.S, Rehman H.U, Usman M (2020). Effect of single or combined supplementation of zinc and probiotics on muscle and bone characteristics and haematobiochemical profile in broilers. Vet. Med.

[69] Zheng Q, Zhang Y, Chen Y, Yang N, Wang X.J, Zhu D (2009). Systematic identification of genes involved in divergent skeletal muscle growth rates of broiler and layer chickens. BMC Genomics.

[70] Clark D.L, Velleman S.G (2016). Spatial influence on breast muscle morphological structure, myofiber size, and gene expression associated with the wooden breast myopathy in broilers. Poult. Sci.

[71] Clark D.L, Walter K.G, Velleman S.G (2017). Incubation temperature and time of hatch impact broiler muscle growth and morphology. Poult. Sci.

[72] Lokman I.H, Jawad H.S.A, Goh Y.M, Sazili A.Q, Noordin M.M, Zuki A.B.Z (2016). Morphology of breast and thigh muscles of red jungle fowl (*Gallus gallus spadiceus*), Malaysian village chicken (*Gallus gallus domesticus*) and commercial broiler chicken. Int. J. Poult. Sci.

[73] Herbst K.L, Bhasin S (2004). Testosterone action on skeletal muscle. Curr. Opin. Clin. Nutr. Metab. Care.

[74] Serra C, Tangherlini F, Rudy S, Lee D, Toraldo G, Sandor N.L, Zhang A, Jasuja R, Bhasin S (2013). Testosterone improves the regeneration of old and young mouse skeletal muscle. J. Gerontol. A Biol. Sci. Med. Sci.

[75] Sinha-Hikim I, Cornford M, Gaytan H, Lee M.L, Bhasin S (2006). Effects of testosterone supplementation on skeletal muscle fiber hypertrophy and satellite cells in community-dwelling older men. J. Clin. Endocrinol. Metab.

[76] Glass D.J (2010). PI3 kinase regulation of skeletal muscle hypertrophy and atrophy. Curr. Top. Microbiol. Immunol.

[77] Adelakun S.A, Ogunlade B, Fidelis O.P, Omotoso O.D (2022). Protective effect of nutritional supplementation of zinc-sulfate against cisplatin-induced spermatogonial and testicular dysfunctions in adult male Sprague-Dawley rats. Endocr. Metab. Sci.

[78] Babaei H, Derakhshanfar A, Kheradmand A, Bazi J (2007). Zinc modulates heat-induced degenerative effects in mice testes. Iran. J. Vet. Res.

[79] Cholifah S, Arsyad A, Salni S (2014). Effect of giving bitter gourd extract (*Momordica charantia* L.) on the histological structure of the testes and epididymis of male rats (*Rattus norvegicus*) Sprague Dawley®. Sriwijaya Med. Mag.

[80] Wongkar J, Durry M.F, Kairupan C.F (2014). Effects of anabolic androgenic steroid administration of low doses and high doses on testicular morphological features wistar (*Rattus norvegicus*). EBiomed.

[81] Ježek D, Šimunić-Banek L, Pezerović-Panijan R (1993). Effects of high doses of testosterone propionate and testosterone enanthate on rat seminiferous tubules-a stereological and cytological study. Arch. Toxicol.

[82] Yama O.E, Duru F.I, Oremosu A.A, Noronha C, Abayomi O (2011). Stereological evaluation of the effects of *Momordica charantia*, antioxidants and testosterone on seminiferous tubules of rat. Int. J. Morphol.

[83] McLachlan R.I, O'Donnell L, Stanton P.G, Balourdos G, Frydenberg M, de Kretser D.M, Robertson D.M (2002). Effects of testosterone plus medroxyprogesterone acetate on semen quality, reproductive hormones, and germ cell populations in normal young men. J. Clin. Endocrinol. Metab.

